# The TBX18/SIX1 Transcriptional Circuit Maintains Stemness and EMT States to Promote Radioresistance in ESCC

**DOI:** 10.3390/cancers18162700

**Published:** 2026-08-20

**Authors:** Liming Gu, Tianqi Yang, Jinmeng Zhang, Jia Wu, Qiang Fan, Yunxia Zhang, Jun Che, Jun Zhu, Ke Gu, Jialiang Zhou

**Affiliations:** Affiliated Hospital of Jiangnan University, Wuxi 214122, China

**Keywords:** ESCC, radioresistance, TBX18, SIX1, positive feedback loop

## Abstract

**Background**: As a member of the T-box transcription factor family, TBX18 was found to be involved in ESCC progression, while its role in regulating radiotherapy resistance in ESCC remains unclear. This study was designed to investigate the molecular mechanisms underlying the regulation of radioresistance in ESCC by TBX18. **Methods**: Sphere formation assay, Transwell invasion assay, and wound healing assay were conducted to show the influence of TBX18 on tumor stemness and epithelial–mesenchymal transition (EMT). Western blot, immunofluorescence, chromatin immunoprecipitation-qPCR (ChIP-qPCR) and dual-luciferase reporter assay were preformed to identify regulatory networks. A nude mouse xenograft tumor model was established to assess the regulatory effect of TBX18 and SIX1 on radioresistance of ESCC in vivo. **Results**: TBX18 expression was positively associated with stemness markers, including CD44, CD271, and SOX2. TBX18 promoted stemness-associated phenotypes, EMT, migration, invasion, and radioresistance in ESCC cells. Mechanistically, TBX18 directly bound to the SIX1 promoter and transcriptionally activated SIX1 expression. In turn, SIX1 enhanced TBX18 protein stability by suppressing ubiquitin–proteasome-mediated degradation, thereby forming a positive feedback loop. Functional rescue experiments demonstrated that the TBX18/SIX1 axis coordinately maintained stemness and EMT phenotypes and attenuated radiotherapy-induced apoptosis. In vivo studies further confirmed that TBX18 knockdown enhanced radiosensitivity, whereas SIX1 overexpression partially reversed this effect. In addition, immunohistochemical analysis revealed that TBX18 and SIX1 were significantly upregulated in ESCC tissues and positively correlated with each other.

## Simple Summary

Radiotherapy is an important radical treatment method for esophageal squamous cell carcinoma (ESCC), effects of which depend on the radiosensitivity of ESCC. In this study, we found that TBX18 promoted ESCC radioresistance through formation of a TBX18/SIX1 positive feedback loop that sustains stemness and EMT programs. Targeting the TBX18/SIX1 axis may represent a potential therapeutic strategy for overcoming ESCC radioresistance.

## 1. Introduction

ESCC is one of the common malignant tumors globally, and its incidence and mortality rates rank among the top in digestive system tumors [1,2]. Radiotherapy is an important radical treatment method or a component of comprehensive treatment for ESCC. However, approximately 50% of patients experience local recurrence or distant metastasis due to radiotherapy resistance [3,4]. The mechanisms of radiotherapy resistance are complex, involving abnormal DNA damage repair, resistance to apoptosis, remodeling of the tumor microenvironment, etc. Among them, the maintenance of tumor cell stemness and epithelial–mesenchymal transition (EMT) have been confirmed as key driving factors [5,6]. Cancer stem cells (CSCs) maintain their self-renewal ability by highly expressing stemness markers such as CD44 and SOX2, while EMT enhances cell migration and invasion abilities by inducing a mesenchymal phenotype. Both are significantly negatively correlated with radiotherapy sensitivity. Therefore, in-depth analysis of the molecular network regulating stemness and EMT is of great clinical significance for developing new strategies to reverse radiotherapy resistance in ESCC.

TBX18 is a member of the T-box transcription factor family, and its function is highly tissue-specific. During embryonic development, TBX18 plays essential roles in cardiovascular development and somite formation [7,8]. Beyond its developmental functions, accumulating evidence has demonstrated that TBX18 is also involved in tumor progression. For example, Su et al. identified TBX18 as a potential regulator of breast cancer progression [9]. In addition, genetic alterations of TBX18 have been reported in prostate cancer and may contribute to disease progression [10]. Our previous study further demonstrated that TBX18 promotes ESCC progression [11]. However, the molecular mechanisms by which TBX18 regulates malignant phenotypes, particularly its role in radiotherapy resistance, remain largely unclear. Given that transcription factors can regulate tumor cell plasticity and therapy response through downstream target genes, TBX18 may influence ESCC radiosensitivity by modulating key biological processes associated with treatment resistance. Considering the critical roles of cancer stemness and epithelial–mesenchymal transition (EMT) in radiotherapy resistance, we hypothesized that TBX18 may contribute to ESCC radioresistance through regulation of these processes.

As a transcription factor of the Homeobox family, SIX1 is abnormally activated in various tumors and promotes tumor progression by regulating EMT, stemness, and chemoresistance [12,13]. For example, SIX1 induces EMT by activating ZEB1 in colorectal cancer [14] and enhances cisplatin resistance by maintaining the cancer stem cell subpopulation in ovarian cancer [15]. Notably, bioinformatics analysis shows a highly positive correlation between the expressions of TBX18 and SIX1 in ESCC (preliminary experimental data of this study), suggesting a possible functional synergy between the two. However, the interaction mechanisms between transcription factors (such as transcriptional activation or protein–protein interaction) have not been reported in ESCC.

This study systematically investigated the role and molecular mechanism of the TBX18/SIX1 axis in ESCC radioresistance through bioinformatics analyses, cellular and molecular experiments, and in vivo models. Our findings reveal a novel transcriptional positive feedback circuit involved in radiotherapy resistance and uncover previously unrecognized functions of the TBX18/SIX1 signaling axis in regulating stemness and EMT in ESCC. These results provide a potential therapeutic target for radiosensitization and offer new insights into the integrated regulatory network linking stemness, EMT, and transcriptional regulation in solid tumors.

## 2. Materials and Methods

### 2.1. Cell Lines and Culture Conditions

The human ESCC cell line KYSE150 was purchased from the Cell Bank of the Chinese Academy of Sciences. Cells were cultured in RPMI 1640 medium supplemented with 10% fetal bovine serum (FBS, Gibco, Grand Island, NY, USA), 100 U/mL penicillin, and 100 μg/mL streptomycin, maintained in a 37 °C incubator with 5% CO_2_.

### 2.2. Primary Reagents and Antibodies

Small Molecules: TGF-β (10 ng/mL, PeproTech, Rocky Hill, NJ, USA) and GB201 (10 μM, Selleck Chemicals, Houston, TX, USA).

Molecular Biology Reagents: TRIzol reagent (Invitrogen, Carlsbad, CA, USA), reverse transcription kit (TaKaRa Bio, Shiga, Japan), SYBR Green PCR Master Mix (Roche, Basel, Switzerland), Lipofectamine 3000 transfection reagent (Invitrogen), and Matrigel (Corning Inc., Corning, NY, USA).

Antibodies: TBX18 (1:1000, Proteintech, Rosemont, IL, USA, 23237-1-AP), SIX1 (1:1000, Proteintech, Rosemont, IL, USA, 10709-1-AP), CD44 (1:1000, BD Biosciences, San Jose, CA, USA, 550538), CD271 (1:1000, Proteintech, Rosemont, IL, USA, 55014-1-AP), SOX2 (1:1000, MilliporeSigma, Burlington, MA, USA, AB5603), E-cadherin (1:1000, Cell Signaling Technology, Danvers, MA, USA, 3195S), N-cadherin (1:1000, Cell Signaling Technology, Danvers, MA, USA, 13116S), Vimentin (1:1000, Abcam, Cambridge, UK, ab92547), and GAPDH (1:5000, Proteintech, Rosemont, IL, USA, 60004-1-Ig).

Plasmids and Viral Vectors: TBX18 overexpression plasmid (pcDNA3.1-TBX18), SIX1 overexpression plasmid (pcDNA3.1-SIX1), TBX18 shRNA (sh-TBX18#1/#2), and SIX1 shRNA (sh-SIX1#1/#2) were constructed by GeneChem (Shanghai Genechem Co., Ltd. China). Lentiviral packaging utilized psPAX2 and pMD2.G plasmids (Addgene, Watertown, MA, USA).

### 2.3. Bioinformatics Analysis

GEPIA2 Database: Analyzed the expression correlation between TBX18 and stemness markers (CD44, CD271, SOX2, CD133, and OCT4) in TCGA-ESCC data using Pearson correlation analysis (*p* < 0.05 was considered significant).

JASPAR Database: Predicted potential binding sites of TBX18 in the SIX1 promoter region (MA0517.1).

### 2.4. Cell Transfection and Establishment of TBX18/SIX1-Modified ESCC Cell Models

Plasmids or siRNA were transfected into ESCC cells using Lipofectamine 3000, and cells were collected for functional assays 48 h later. For lentiviral infection, ESCC cells were selected with puromycin (2 μg/mL) for 2 weeks after infection. The efficiency of TBX18 knockdown and TBX18 overexpression was verified by Western blot analysis, and monoclonal cell lines were selected for subsequent experiments.

### 2.5. Western Blot Analysis for Validation of TBX18/SIX1 Expression and Downstream Signaling Proteins

Total proteins were extracted from cell lysates using RIPA buffer, and protein concentration was determined by BCA assay. Proteins were separated by 10% SDS-PAGE and transferred to PVDF membranes (Millipore). Membranes were blocked with 5% skimmed milk for 1 h, incubated with primary antibodies at 4 °C overnight, followed by HRP-conjugated secondary antibodies (1:5000) at room temperature for 1 h. Protein bands were visualized using ECL chemiluminescent reagents (Millipore).

### 2.6. Immunofluorescence Staining and Immunohistochemistry Staining

Cells were seeded onto coverslips in 24-well plates, fixed with 4% paraformaldehyde for 15 min, permeabilized with 0.5% Triton X-100 for 10 min, and blocked with 5% BSA for 30 min. After overnight incubation with primary antibodies at 4 °C, cells were stained with Alexa Fluor 488/594-conjugated secondary antibodies (1:500, Invitrogen) at room temperature for 1 h, counterstained with DAPI for 5 min, and visualized by laser confocal microscopy.

Paraffin-embedded ESCC tissue sections and adjacent normal tissue sections were subjected to immunohistochemical staining. Briefly, tissue sections were deparaffinized, rehydrated, and subjected to antigen retrieval. After blocking endogenous peroxidase activity and nonspecific binding, sections were incubated with primary antibodies against TBX18, SIX1, and Ki-67 overnight at 4 °C, followed by incubation with HRP-conjugated secondary antibodies. Immunoreactive signals were visualized using a DAB substrate kit and counterstained with hematoxylin.

The immunohistochemical staining results were independently evaluated by two experienced pathologists who were blinded to the clinical information. The staining intensity and the percentage of positively stained tumor cells were assessed using a semi-quantitative scoring system. Any discrepancies between the two observers were resolved through discussion and consensus.

### 2.7. Sphere Formation Assay for Evaluating Tumor Stemness-Associated Self-Renewal Ability

Single-cell suspensions (1 × 10^3^ cells/well) were seeded into ultra-low attachment 6-well plates with serum-free medium containing 20 ng/mL EGF, 10 ng/mL bFGF, and B27 (1:50, Gibco). After 7 days of culture, spheres with diameters > 50 μm were counted under a microscope. Each group included 3 replicate wells, and the experiment was repeated 3 times.

### 2.8. Transwell Migration and Invasion Assays for Evaluating Metastatic Potential

Invasion Assay: The upper chamber was coated with Matrigel (1:8 dilution, Corning Inc., Corning, NY, USA), and 2 × 10^4^ cells in serum-free medium were seeded. The lower chamber contained medium with 10% FBS. After 24 h, invasive cells were stained with crystal violet and counted.

Wound Healing Assay: When cells reached 90% confluence, scratches were created using a 200 μL pipette tip. After washing with PBS, cells were cultured in serum-free medium. Images were captured at 0 and 24 h using an inverted microscope (IX73; Olympus Corporation, Tokyo, Japan), and scratch widths were measured using ImageJ software (version 1.54g; National Institutes of Health, Bethesda, MD, USA).

### 2.9. Chromatin Immunoprecipitation Assay for Validation of TBX18 Binding to the SIX1 Promoter

Following the protocol of the ChIP kit (Millipore, 17-372), cells were cross-linked with 1% formaldehyde, and chromatin was fragmented by sonication (200–1000 bp). Ten percent of the lysate was saved as Input, and the remaining lysate was incubated with TBX18 antibody (5 μg) or IgG control at 4 °C overnight. After binding to Protein A/G magnetic beads, DNA was extracted and used for qPCR, which was performed to detect the enrichment of the SIX1 promoter fragment (NC_000014.9: c60651477-60649477, GRCh38.p14 Primary Assembly) containing the predicted TBX18-binding region (Region: upstream primer 5′-CACTCGAGTGATGTTTATTTTT-3′, downstream primer 5′-CTTCGTTTTCAGCCAAGAGG-3′).

### 2.10. Co-Immunoprecipitation (Co-IP)

Cell lysates were prepared with 1% NP-40 lysis buffer. Five hundred micrograms of total protein was incubated with TBX18 antibody or IgG control at 4 °C for 2 h, followed by Protein A/G magnetic beads overnight. After washing, beads were boiled for denaturation, and co-precipitated SIX1 protein was detected by Western blot.

### 2.11. Xenograft Tumor Model for Evaluating TBX18/SIX1-Mediated Tumor Growth and Radiotherapy Response

All animal procedures were approved by the Institutional Animal Care and Use Committee (IACUC) of Yi Shengyuan Gene Technology (Tianjin, China) Co., Ltd. and performed in accordance with institutional and national guidelines (Animal Ethics Approval Number: YSY-DWLL-2024823). Prior to tumor irradiation, mice were anesthetized with isoflurane (5% for induction and 2% for maintenance) using a precision vaporizer. At the experimental endpoint, mice were euthanized under deep anesthesia with isoflurane (5% induction) followed by cervical dislocation, an IACUC-approved method ensuring rapid and humane death.

Six-week-old female BALB/c nude mice (SPF grade, Shanghai SLAC Laboratory Animal Center, Shanghai, China) were randomly divided into three groups (*n* = 6/group): sh-NC group, sh-TBX18#2 group, and sh-TBX18#2+SIX1 group. KYSE150 ESCC cells stably expressing the indicated constructs were prepared and subcutaneously inoculated into the right axilla of nude mice (5 × 10^6^ cells per mouse). Each group was subcutaneously inoculated with 5 × 10^6^ cells in PBS. When tumor volume reached 80 mm^3^, mice received local single-dose radiotherapy (6 Gy, X-ray irradiator, Precision X-Ray). Tumor volume was measured every 3 days (volume = 0.5 × length × width^2^). Mice were sacrificed after 21 days, and tumors were dissected and weighed. Tumor tissues were fixed with 4% paraformaldehyde for HE staining and immunohistochemical (IHC) detection (Ki-67, TBX18, and SIX1 antibodies, 1:200 dilution).

### 2.12. Flow Cytometry for Apoptosis Detection

Treated cells were collected by trypsinization, stained with Annexin V-FITC/PI (BD Biosciences, San Jose, CA, USA), and analyzed by flow cytometry (BD Biosciences, San Jose, CA, USA FACSCanto II). Apoptotic cell percentages were quantified using FlowJo software (FlowJo 11).

### 2.13. Luciferase Reporter Assay for Validation of TBX18-Mediated SIX1 Transcriptional Regulation

The putative TBX18-binding region within the SIX1 promoter was predicted using the JASPAR database. The human SIX1 promoter fragment (2001 bp; GRCh38.p14 Primary Assembly, NC_000014.9: c60651477-60649477) containing the predicted TBX18-binding motif was amplified and cloned into the pGL4.22[luc2CP/Puro] luciferase reporter vector. The promoter sequence information is provided in Table S1. A mutant reporter plasmid was generated by introducing point mutations into the predicted core TBX18-binding motif.

For the overexpression assay, ESCC cells were co-transfected with wild-type or mutant SIX1 promoter reporter plasmids containing the predicted TBX18-binding motif in Region 2. The wild-type motif sequence (5′-AGGTGGGAA-3′) was mutated to 5′-AGGAGAGAA-3′ by introducing two nucleotide substitutions to disrupt the predicted TBX18-binding site. The reporter plasmids were co-transfected with either the TBX18 overexpression plasmid or empty vector control. For the knockdown assay, cells were co-transfected with the reporter plasmids and sh-TBX18 or sh-NC vectors. A Renilla luciferase plasmid was co-transfected as an internal control to normalize transfection efficiency. After 24–48 h of transfection, cells were harvested and luciferase activity was measured using a dual-luciferase reporter assay system according to the manufacturer’s instructions (Promega, Madison, WI, USA; Cat. No. E1910). Firefly luciferase activity was normalized to Renilla luciferase activity. Relative luciferase activity was expressed as fold change compared with the corresponding control group.

### 2.14. Statistical Analysis

Experimental data are presented as mean ± standard deviation (Mean ± SD). Student’s *t*-test (for two groups) or one-way analysis of variance (ANOVA, for multiple groups) was performed using GraphPad Prism 9 software. *p* < 0.05 was considered statistically significant.

## 3. Results

### 3.1. TBX18 Promotes the Stemness of ESCC Cells

Cellular stemness is considered an important factor influencing tumorigenesis, development, and treatment resistance [16,17]. In our previous study, we have confirmed that TBX18 can promote the radiotherapy tolerance of ESCC, but its specific molecular mechanism remains unclear [11]. Given the crucial role of tumor stemness in radiation resistance [18], we initially explored the potential association between TBX18 and tumor stemness.

First, we utilized the GEPIA2 database to analyze the expression correlation between TBX18 and a series of tumor stemness-related markers (including CD44, CD271, SOX2, CD133, and OCT4). The results showed that TBX18 was significantly positively correlated with CD44, CD271, and SOX2 (Figure S1A–E), suggesting that TBX18 may be closely related to tumor stemness. To further verify this hypothesis, we constructed ESCC cell lines with stable knockdown or overexpression of TBX18 and confirmed the intervention efficiency by Western blot. The results showed that sh-TBX18#2 had the best knockdown efficiency, and the protein level in TBX18-overexpressing cells increased significantly (Figure S1F).

**Figure 1:**
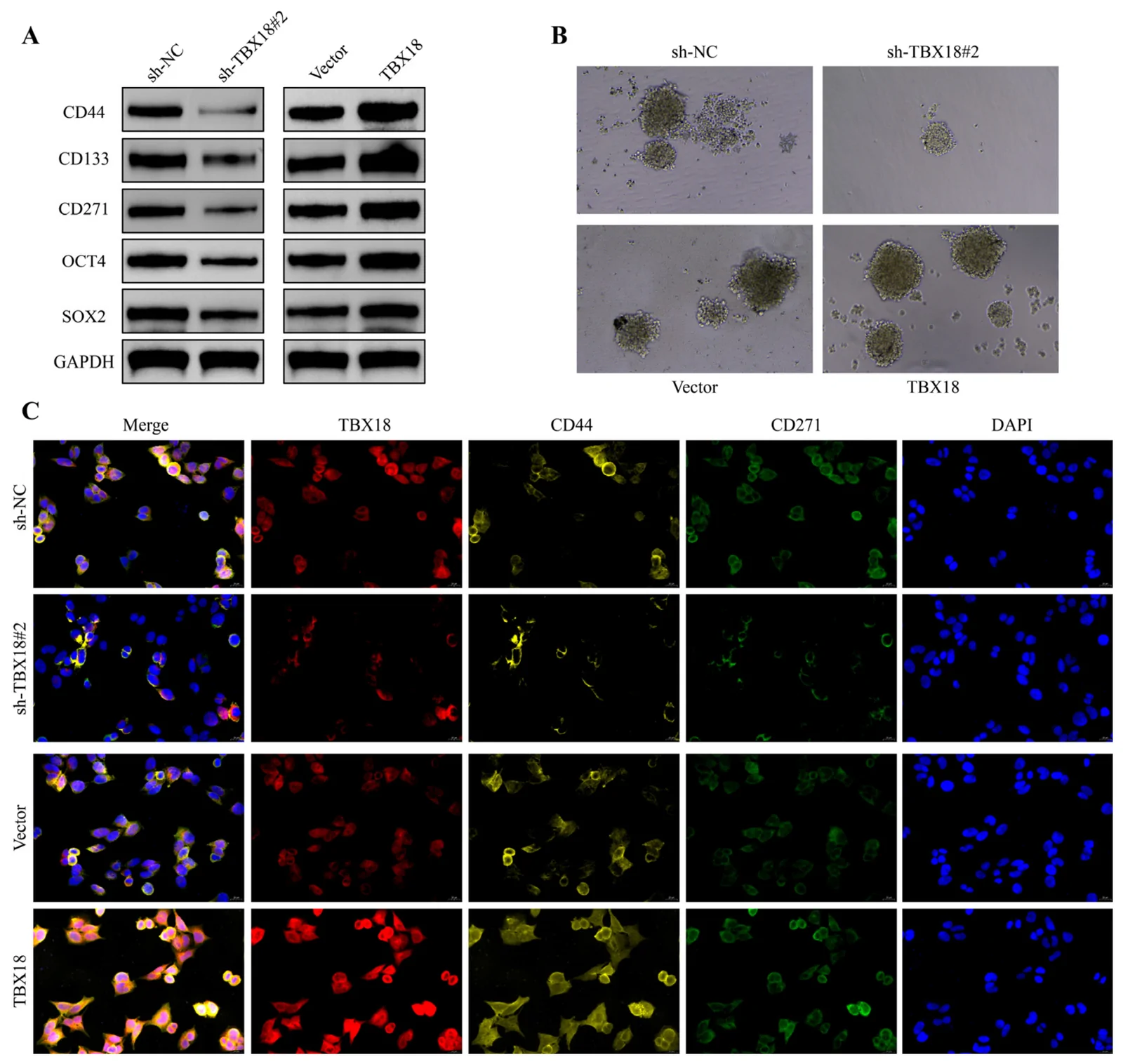
TBX18 regulates stemness in esophageal squamous cell carcinoma (ESCC) cells. (**A**) Western blot analysis showing the protein levels of stemness-associated markers (CD44, CD133, CD271, OCT4, and SOX2) after TBX18 knockdown (sh-TBX18#2) or overexpression (TBX18). (**B**) Representative images of sphere formation assays evaluating the stemness-associated tumorsphere-forming capacity of ESCC cells after TBX18 manipulation. (**C**) Immunofluorescence staining for TBX18 (red), CD44 (yellow), and CD271 (green) in ESCC cells following TBX18 knockdown or overexpression. Nuclei were counterstained with DAPI (blue). Scale bar, 20 μm.

Subsequently, we detected the expression changes of stemness-related markers. The results indicated that knocking down TBX18 could significantly downregulate the protein levels of CD44, CD271, SOX2, CD133, and OCT4; conversely, overexpressing TBX18 could upregulate the expression of the above stemness markers (Figure 1A). The sphere formation assay further demonstrated that TBX18 significantly enhanced the tumorsphere-forming ability of ESCC cells, suggesting that TBX18 promotes stemness-associated properties (Figure 1B). Consistently, Western blot analysis showed that TBX18 knockdown reduced, whereas TBX18 overexpression increased, the expression of stemness-associated markers, including CD44, CD271, SOX2, CD133, and OCT4 (Figure 1C). These findings indicate that TBX18 promotes stemness-associated phenotypes in ESCC cells.

### 3.2. TBX18 Promotes the Migration, Invasion, and Radioresistance of ESCC Cells by Regulating the EMT Process

**Figure 2:**
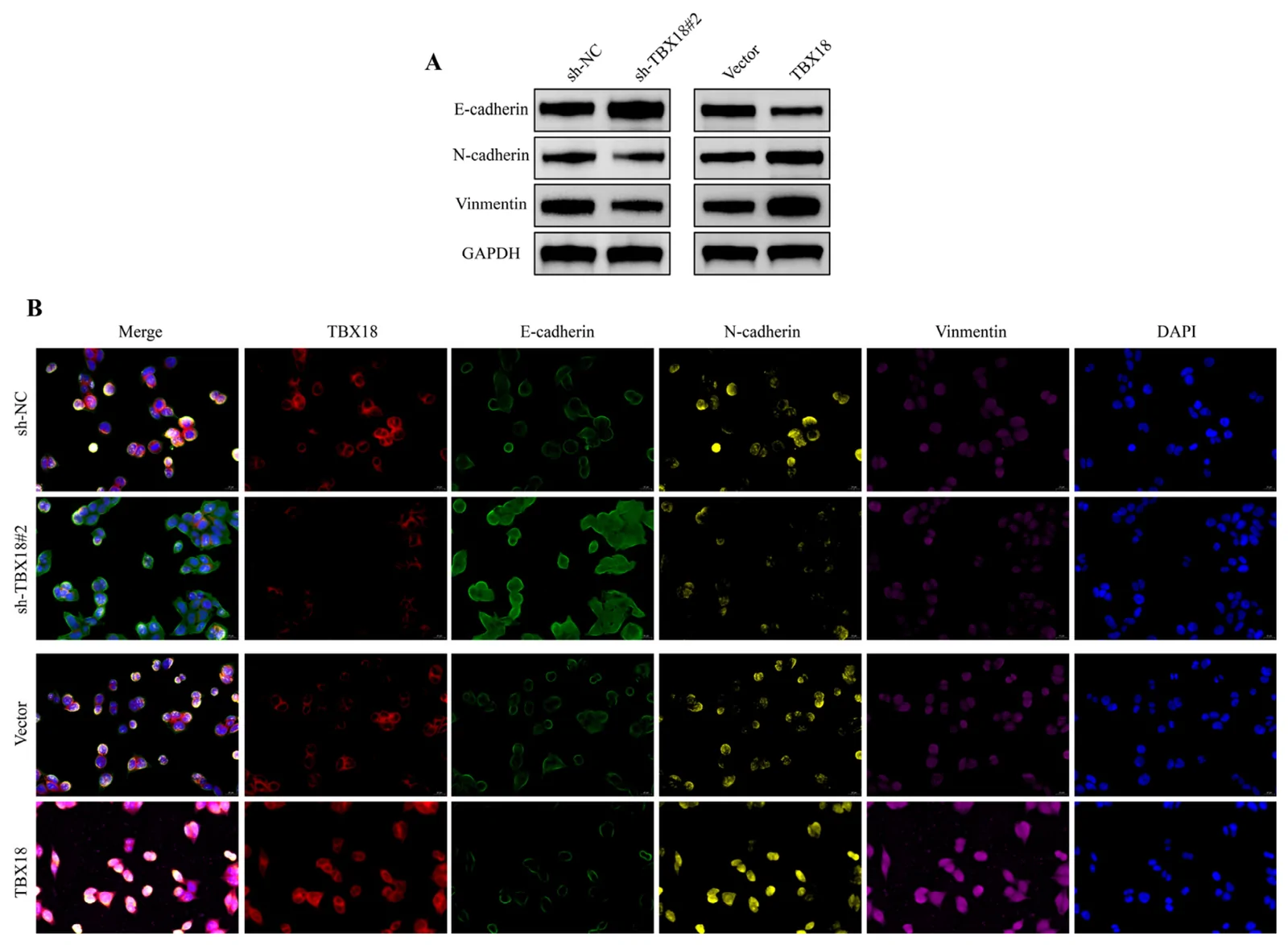
TBX18 promotes epithelial–mesenchymal transition (EMT) in ESCC cells. (**A**) Western blot analysis of EMT-related markers in ESCC cells after TBX18 knockdown or overexpression. (**B**) Immunofluorescence staining showing the expression and localization of TBX18 (red), E-cadherin (green), N-cadherin (yellow), and Vimentin (purple) in ESCC cells. Nuclei were counterstained with DAPI (blue). Scale bar, 20 μm.

EMT plays a crucial role in the invasion and metastasis of tumor cells [19]. Previous studies have shown that EMT not only promotes the chemoresistance of various tumors but also reduces the sensitivity of ESCC to radiotherapy [20,21]. To investigate whether TBX18 is involved in the EMT transformation process of ESCC cells, we first performed Transwell invasion assay and wound healing assay. The results showed that knocking down TBX18 could significantly inhibit the migration and invasion abilities of ESCC cells, while overexpressing TBX18 significantly enhanced these abilities (Figure S2A,B), suggesting its possible involvement in EMT regulation. Furthermore, we used Western blot and immunofluorescence analysis to detect the changes in EMT-related markers. The results showed that, with the knockdown or overexpression of TBX18, the mesenchymal phenotype markers N-cadherin and Vimentin showed a downward or upward trend, respectively, while the epithelial phenotype marker E-cadherin showed the opposite change (Figure 2A,B), indicating that TBX18 may regulate the EMT state of ESCC cells.

To further verify the importance of EMT in the mediating role of TBX18, we added the EMT activator TGF-β for intervention. The results showed that TGF-β could reverse the EMT phenotype changes caused by knocking down TBX18 (Figure S3A,B), further proving that TBX18 has a certain functional dependence on EMT regulation. In summary, TBX18 may enhance the migration and invasion abilities of ESCC cells and reduce their sensitivity to radiotherapy to a certain extent by inducing the EMT process.

### 3.3. TBX18 Affects the Sensitivity of ESCC Cells to Radiotherapy by Regulating Tumor Stemness and EMT Simultaneously

**Figure 3:**
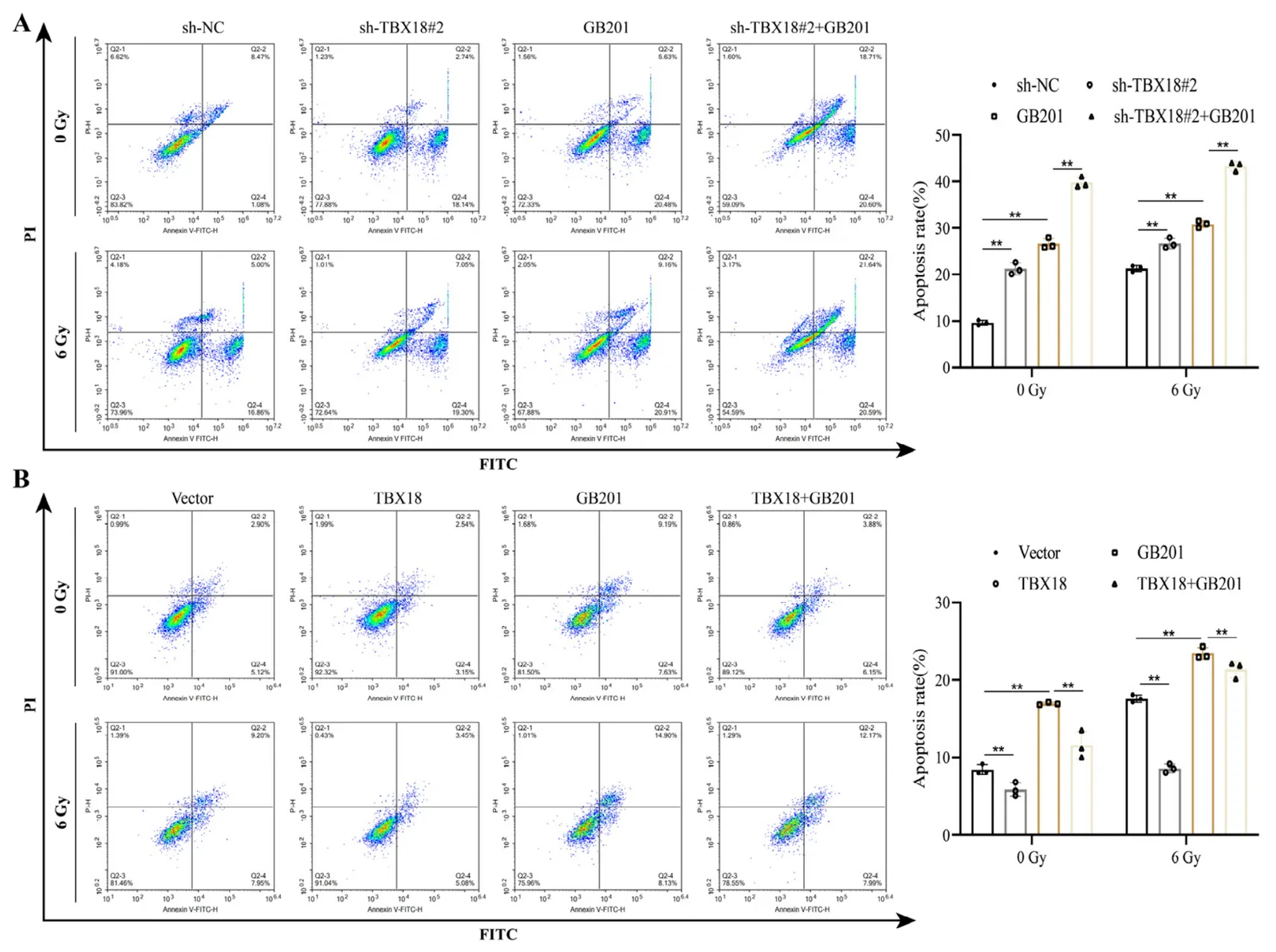
TBX18 modulates radiosensitivity and apoptosis in ESCC cells. (**A**) Flow cytometric analysis of apoptosis after TBX18 knockdown (sh-TBX18#2), with or without the stemness-associated pathway inhibitor GB201. Cells were untreated (0 Gy) or irradiated (6 Gy). Representative plots (left) and quantification of apoptosis (right) are shown. (**B**) Flow cytometric analysis of apoptosis after TBX18 overexpression, with or without GB201. TBX18 overexpression reduced irradiation-induced apoptosis, while GB201 partially reversed this effect. Data are mean ± SD from three independent experiments. ** *p* < 0.01.

To further investigate whether TBX18 mediates the radioresistance of ESCC cells by regulating tumor stemness and the EMT process, we introduced the small molecule inhibitor GB201 targeting the stemness pathway for functional verification. GB201 is a broad-spectrum and efficient cancer stem cell pathway inhibitor that can significantly inhibit the self-renewal and survival of tumor stem cells and has definite anti-tumor activity [22,23]. The experimental results showed that combined treatment with GB201 on the basis of knocking down TBX18 expression could further enhance the apoptosis level of ESCC cells compared with knocking down TBX18 alone (Figure 3A). Conversely, when GB201 was used in combination with TBX18 overexpression, although it could still promote cell apoptosis, the apoptosis level was lower than that in the GB201 single-treatment group (Figure 3B). This result suggests that TBX18 may enhance the radioresistance by strengthening the stemness of tumor cells and weakening the pro-apoptotic effect of GB201. Interestingly, when the EMT activator TGF-β was introduced, we observed that the pro-apoptotic effect caused by knocking down TBX18 was partially reversed (Figure S4A), and the apoptosis inhibitory effect caused by TBX18 overexpression was further enhanced (Figure S4B). This further suggests that the EMT process also plays a crucial role in TBX18-mediated radioresistance. In conclusion, the above results indicate that TBX18 affects the sensitivity of ESCC cells to radiotherapy by regulating tumor stemness and the EMT process simultaneously, and its radioresistance mechanism has the characteristic of multi-pathway coordination.

### 3.4. TBX18 Transcriptionally Activates SIX1 and Forms a Positive Feedback Circuit with SIX1 in ESCC Cells

**Figure 4:**
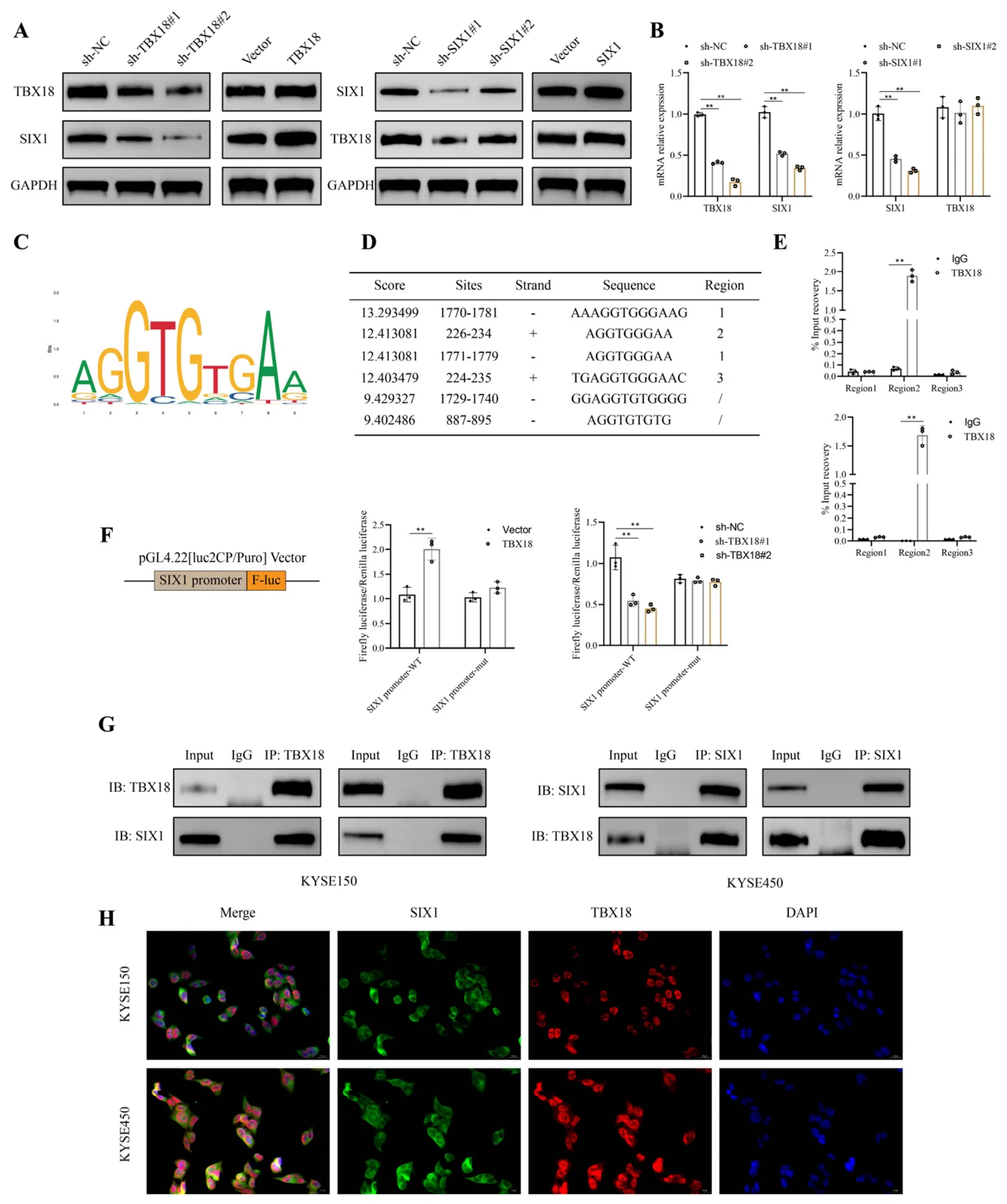
TBX18 transcriptionally activates SIX1 and forms a positive feedback circuit with SIX1 in ESCC cells. (**A**) Western blot analysis of TBX18 and SIX1 expression after TBX18 or SIX1 knockdown/overexpression. (**B**) qRT-PCR validation of TBX18 and SIX1 mRNA expression following knockdown. (**C**) Sequence logo of the predicted TBX18-binding motif identified by JASPAR analysis. (**D**) Bioinformatic prediction of putative TBX18-binding regions within the SIX1 promoter. (**E**) ChIP-qPCR analysis showing enrichment of TBX18 occupancy at the predicted Region 2 of the SIX1 promoter in ESCC cells. (**F**) Dual-luciferase reporter assays using wild-type and mutant SIX1 promoter constructs demonstrating that TBX18 regulates SIX1 transcription in a binding motif-dependent manner. Mutation of the predicted TBX18-binding motif largely abolished these effects. (**G**) Reciprocal co-immunoprecipitation (Co-IP) assays confirming the interaction between TBX18 and SIX1 in ESCC cells. The left and right panels represent KYSE150 and KYSE450 cells, respectively. TBX18 was immunoprecipitated and detected by immunoblotting for SIX1, and reciprocal immunoprecipitation of SIX1 was performed to detect TBX18. (**H**) Immunofluorescence staining showing the expression and subcellular localization of TBX18 and SIX1 in ESCC cells. The upper and lower panels represent KYSE150 and KYSE450 cells, respectively. TBX18 and SIX1 were detected using specific antibodies, and nuclei were counterstained with DAPI. Scale bar, 20 μm. Data are mean ± SD from three independent experiments. ** *p* < 0.01.

Previous studies have shown that SIX1 can also promote stemness maintenance and EMT induction in various tumors [12,24]. Therefore, we speculated that TBX18 may be involved in the radioresistance process of ESCC by regulating SIX1. Since both are transcription factors, the mutual regulatory relationship between them is unclear. We first detected the effect of TBX18 expression changes on SIX1 by Western blot and qPCR. The results showed that knocking down TBX18 could significantly downregulate the protein and mRNA levels of SIX1, while overexpressing TBX18 significantly increased the expression level of SIX1 (Figure 4A,B). Furthermore, we constructed SIX1 knockdown and overexpression cell lines and found that the expression of SIX1 could significantly affect the protein level of TBX18 but not its mRNA level (Figure 4A,B), suggesting that SIX1 may stabilize the expression of TBX18 through protein interaction. To determine whether TBX18 directly regulates SIX1 transcription, we analyzed the SIX1 promoter using the JASPAR transcription factor binding database and identified several putative TBX18-binding motifs within the SIX1 promoter region (Figure 4C,D). ChIP-qPCR analysis further demonstrated a significant enrichment of TBX18 occupancy at Region 2 of the SIX1 promoter, whereas minimal enrichment was observed at the other predicted regions (Figure 4E), indicating preferential binding of TBX18 to this motif-containing promoter region. To further validate the transcriptional regulatory effect of TBX18 on SIX1, we constructed wild-type and motif-mutant SIX1 promoter luciferase reporters. Dual-luciferase reporter assays showed that TBX18 overexpression significantly enhanced the activity of the wild-type SIX1 promoter, whereas mutation of the predicted TBX18-binding motif largely abolished this activation. Consistently, TBX18 knockdown markedly suppressed wild-type promoter activity but exerted minimal effects on the mutant reporter (Figure 4F), indicating that TBX18 transcriptionally activates SIX1 in a motif-dependent manner. In addition, Co-immunoprecipitation assays revealed a direct physical interaction between TBX18 and SIX1 proteins (Figure 4G). Immunofluorescence staining further demonstrated similar nuclear localization patterns of TBX18 and SIX1 in ESCC cells (Figure 4H), supporting their coordinated functional interaction. Collectively, these findings demonstrate that TBX18 directly binds to and transcriptionally activates the SIX1 promoter, while SIX1, in turn, enhances TBX18 protein stability, thereby forming a self-reinforcing positive feedback circuit that may contribute to EMT maintenance and radioresistance in ESCC.

### 3.5. SIX1 Stabilizes TBX18 Protein by Suppressing Ubiquitin–Proteasome-Mediated Degradation

**Figure 5:**
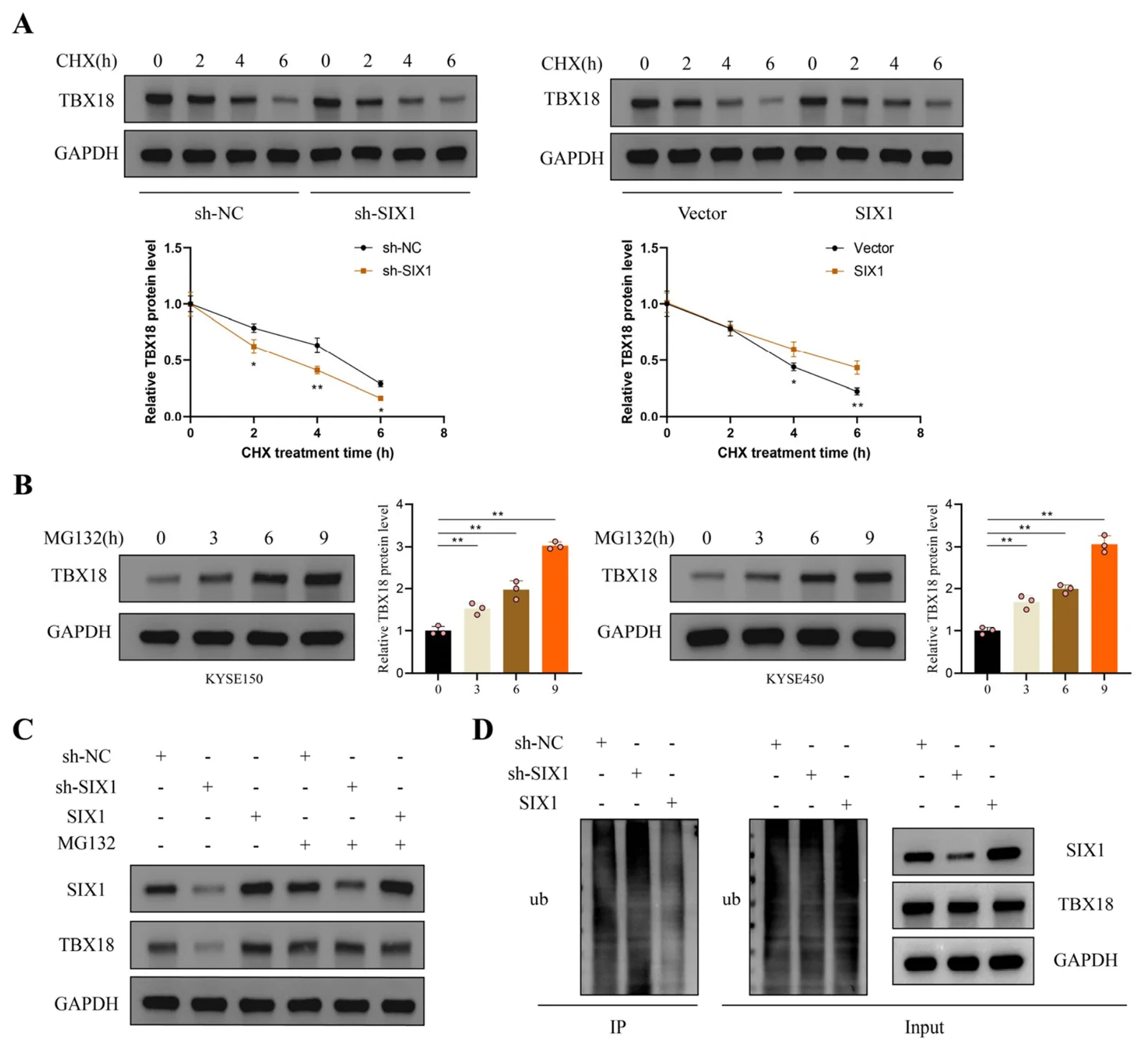
SIX1 stabilizes TBX18 protein expression through suppression of ubiquitin–proteasome-mediated degradation. (**A**) CHX chase assays showing the stability of TBX18 protein after SIX1 knockdown or overexpression in ESCC cells. CHX chase assays showing the effect of SIX1 manipulation on TBX18 protein stability. (**B**) Western blot analysis showing the accumulation of TBX18 protein after MG132 treatment in KYSE150 and KYSE450 cells. Relative TBX18 protein levels were quantified by densitometric analysis and normalized to GAPDH. Data are presented as mean ± SD from three independent experiments. (**C**) Western blot analysis showing that MG132 treatment partially reversed the reduction in TBX18 protein induced by SIX1 knockdown, while SIX1 overexpression further increased TBX18 protein accumulation. (**D**) Ubiquitination assays showing that SIX1 knockdown increased the ubiquitination level of TBX18, whereas SIX1 overexpression reduced TBX18 ubiquitination in ESCC cells. Input controls are shown on the right. * *p* < 0.05; ** *p* < 0.01.

To further clarify how SIX1 regulates TBX18 expression, we investigated whether SIX1 affects TBX18 protein stability. Cycloheximide (CHX) chase assays demonstrated that knockdown of SIX1 markedly accelerated TBX18 protein degradation, whereas ectopic expression of SIX1 significantly prolonged TBX18 protein stability in ESCC cells (Figure 5A). These findings suggested that SIX1 contributes to the maintenance of TBX18 protein abundance. Given that protein degradation is primarily mediated through the ubiquitin–proteasome pathway, we next treated cells with the proteasome inhibitor MG132. MG132 treatment progressively restored TBX18 protein accumulation over time (Figure 5B). Moreover, the reduction in TBX18 induced by SIX1 knockdown was substantially rescued following MG132 treatment, while SIX1 overexpression further enhanced TBX18 accumulation in the presence of MG132 (Figure 5C), indicating that SIX1 regulates TBX18 through a proteasome-dependent mechanism. To further determine whether SIX1 affects TBX18 ubiquitination, ubiquitination assays were performed after modulation of SIX1 expression. Knockdown of SIX1 markedly increased the ubiquitination level of TBX18, whereas overexpression of SIX1 reduced TBX18 ubiquitination (Figure 5D), suggesting that SIX1 protects TBX18 from ubiquitin-mediated proteasomal degradation.

### 3.6. The Positive Feedback Between TBX18 and SIX1 Synergistically Promotes ESCC Radiotherapy Tolerance by Regulating Stemness and EMT

**Figure 6:**
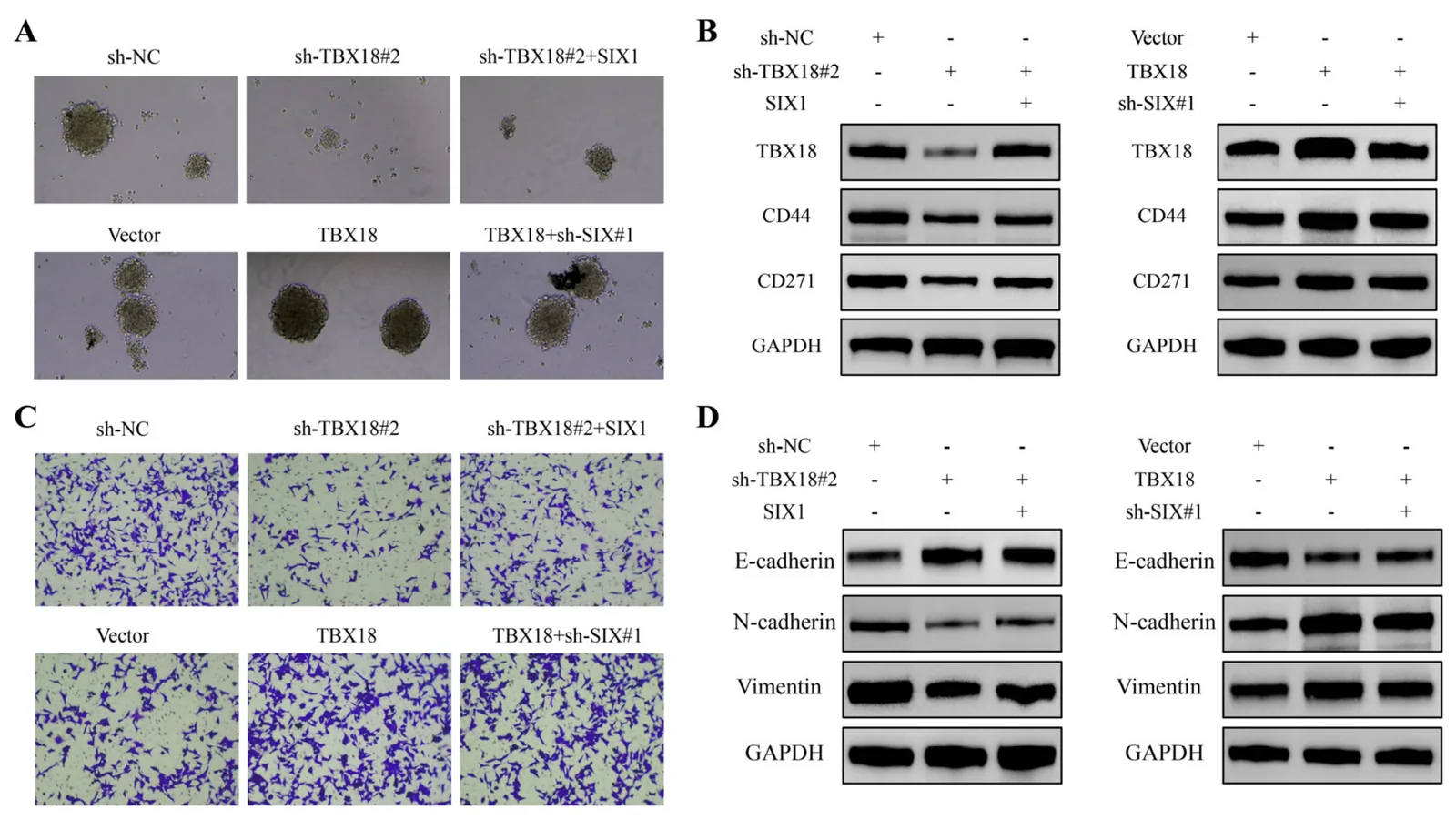
SIX1 mediates TBX18 regulation of stemness, migration, and EMT in ESCC cells. (**A**) Sphere formation assay showing that TBX18 knockdown reduced, whereas TBX18 overexpression enhanced, the sphere-forming capacity of ESCC cells. (**B**) Western blot analysis of stemness-associated markers (CD44 and CD271) after modulation of TBX18 and SIX1 expression. (**C**) Transwell invasion assays demonstrating that SIX1 restored TBX18-regulated migratory and invasive abilities. (**D**) Western blot analysis of EMT markers (E-cadherin, N-cadherin, and Vimentin) showing that SIX1 modulated TBX18-induced EMT changes.

To verify whether the positive feedback loop formed by TBX18 and SIX1 functionally regulates the tumor stemness and EMT processes, we carried out a series of functional rescue experiments. First, using the sphere formation assay and Transwell migration and invasion assay, the results showed that overexpressing SIX1 on the basis of knocking down TBX18 or knocking down SIX1 on the basis of overexpressing TBX18 could partially reverse the phenotypic changes caused by TBX18 single treatment, manifested as the restoration of tumor stemness and migration/invasion abilities (Figure 6A–D). Western blot detection of changes in EMT and stemness markers also supported the above conclusion (Figure 6A–D). Furthermore, in the flow cytometry detection, we found that knocking down TBX18 could significantly promote the apoptosis of ESCC cells, while overexpressing SIX1 at the same time could partially reverse this pro-apoptotic effect.

**Figure 7:**
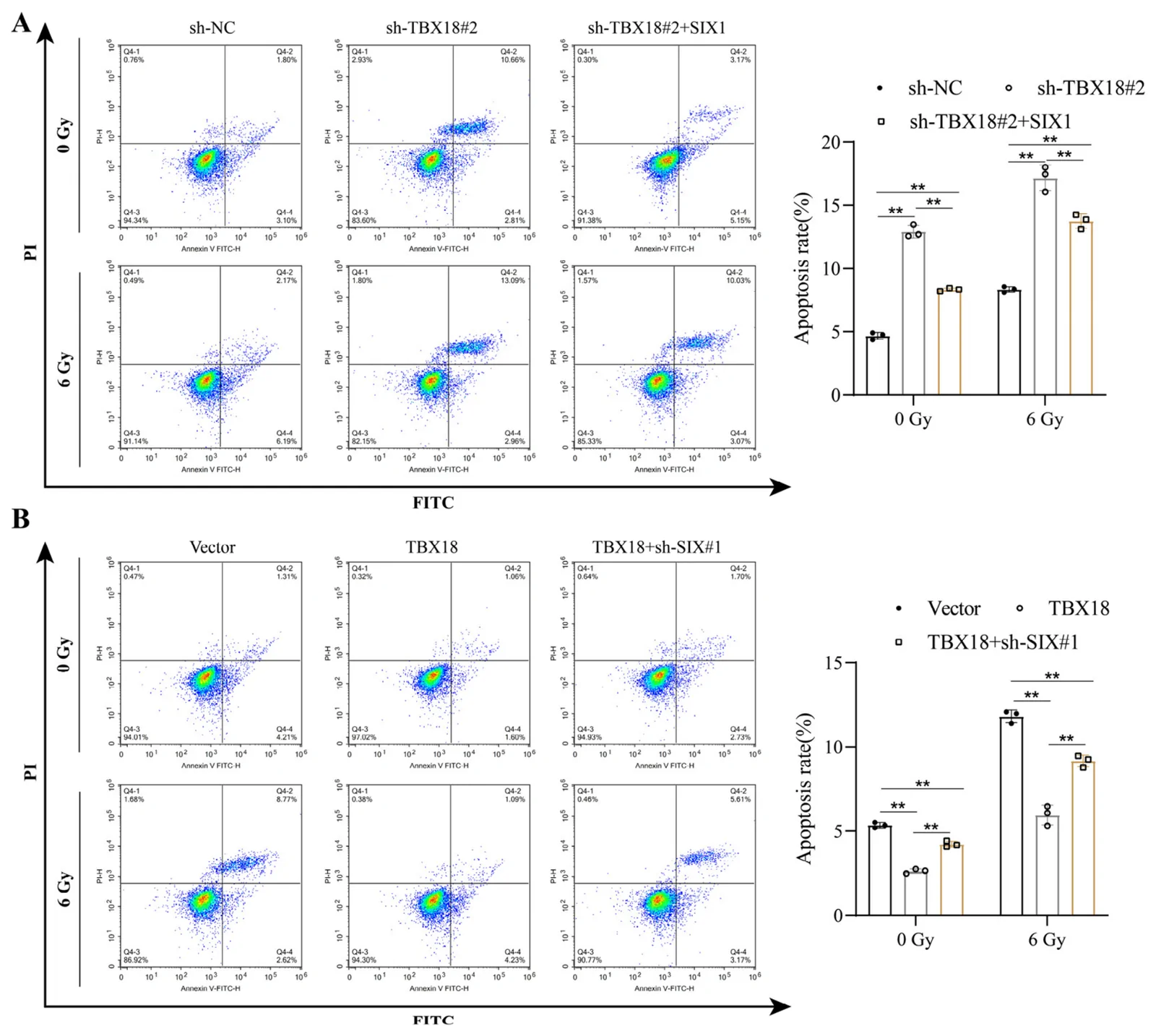
SIX1 mediates the effects of TBX18 on irradiation-induced apoptosis. (**A**) Flow cytometric analysis of apoptosis in ESCC cells after TBX18 knockdown with or without SIX1 overexpression under basal and irradiation (6 Gy) conditions. (**B**) Flow cytometric analysis of apoptosis in TBX18-overexpressing cells with or without SIX1 knockdown, under basal and irradiation (6 Gy) conditions. SIX1 depletion increased irradiation-induced apoptosis and partially abolished TBX18-mediated radioresistance. Data are presented as mean ± SD from three independent experiments. ** *p* < 0.01.

Under the background of TBX18 overexpression, the silencing of SIX1 could also enhance the degree of cell apoptosis (Figure 7A,B). In summary, these experimental results support from the functional level that TBX18 and SIX1 jointly maintain the stemness characteristics and EMT state of tumor cells through a positive feedback mechanism, thereby enhancing the radiotherapy tolerance of ESCC.

### 3.7. The TBX18/SIX1 Positive Feedback Loop Promotes ESCC Radiotherapy Tolerance in a Mouse Xenograft Model

**Figure 8:**
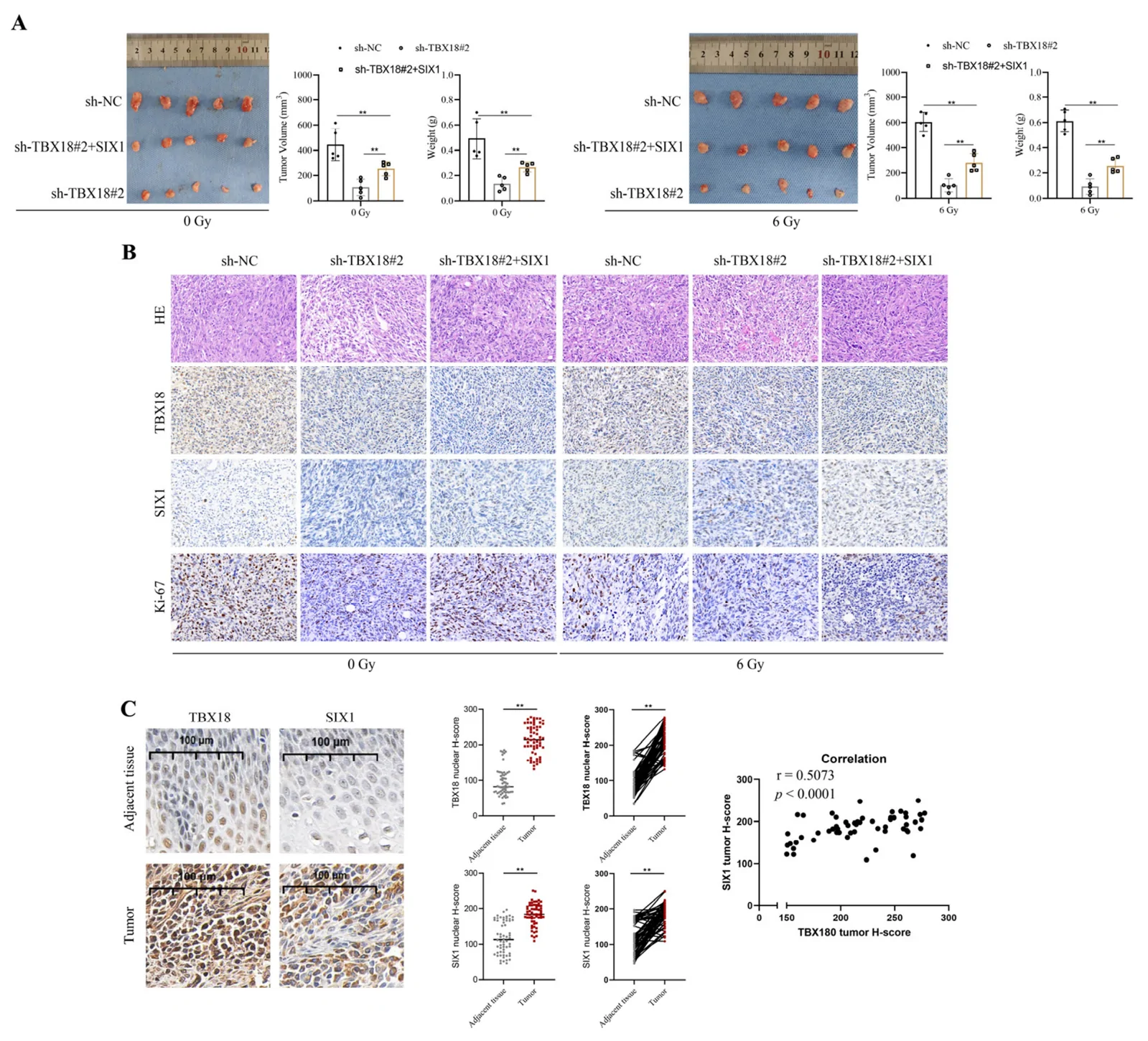
SIX1 mediates TBX18 regulation of tumor growth and radiosensitivity in vivo. (**A**) Nude mouse xenograft model showing the effect of TBX18/SIX1 manipulation on tumor growth after irradiation. Mice were implanted with ESCC cells expressing sh-NC, sh-TBX18, or sh-TBX18 combined with SIX1 overexpression and received local irradiation (6 Gy). Representative tumor images and quantitative analysis of tumor volume and tumor weight are shown. (**B**) Representative H&E staining and immunohistochemical staining of xenograft tumor tissues for TBX18, SIX1, and Ki-67. Changes in TBX18/SIX1 expression and proliferative activity were evaluated among different treatment groups. (**C**) Representative immunohistochemical staining of TBX18 and SIX1 in clinical ESCC tissues and adjacent normal tissues. Nuclear expression levels were quantified using H-score analysis, and the correlation between TBX18 and SIX1 expression was evaluated. ** *p* < 0.01.

To further verify the function of the TBX18-SIX1 positive feedback loop in radioresistance in vivo, we established a nude mouse xenograft tumor model. The following constructed cell lines were inoculated subcutaneously into nude mice: sh-NC (negative control), sh-TBX18#2, and sh-TBX18#2+SIX1. When the tumor volume grew to about 80 mm^3^, local irradiation (6 Gy) was given to simulate radiotherapy intervention. The experimental results showed that, in the irradiated xenograft cohort, TBX18 knockdown significantly enhanced the inhibitory effect of radiotherapy on tumor growth compared with the sh-NC group, whereas SIX1 overexpression partially rescued this phenotype. It is worth noting that, in the sh-TBX18#2+SIX1 group with overexpression of SIX1 on the basis of knocking down TBX18, the tumor volume and weight showed an upward trend compared with the sh-TBX18#2 group, suggesting that SIX1 could partially reverse the tumor inhibitory effect caused by knocking down TBX18 (Figure 8A). To further verify the mechanism, we performed HE staining and IHC detection on the xenograft tumor tissues to evaluate the expression levels of TBX18, SIX1, and Ki-67. The results showed that, under radiotherapy intervention, the expression of Ki-67 and SIX1 in the tumors of the sh-TBX18#2 group decreased significantly, while, in the sh-TBX18#2+SIX1 group, the above indicators were partially restored, which was consistent with the trend of in vitro experiments (Figure 8B). In conclusion, the in vivo experiment further confirms that TBX18 can form a positive feedback regulatory loop by transcriptionally activating SIX1, maintaining the stemness and proliferative ability of tumor cells, thereby reducing the sensitivity of ESCC to radiotherapy.

In addition, analysis of clinical ESCC tissues demonstrated that both TBX18 and SIX1 were significantly upregulated in tumor tissues compared with adjacent normal tissues based on nuclear H-score evaluation. Correlation analysis further revealed a significant positive association between TBX18 and SIX1 expression levels in ESCC specimens (r = 0.5073, *p* < 0.0001) (Figure 8C), supporting the clinical relevance of the TBX18/SIX1 regulatory circuit.

Collectively, these findings demonstrate that TBX18 promotes ESCC radioresistance through formation of a self-reinforcing TBX18/SIX1 positive feedback circuit, thereby sustaining tumor proliferation and malignant progression during radiotherapy.

## 4. Discussion

Radiation tolerance is one of the important causes of treatment failure in esophageal squamous cell carcinoma (ESCC), and its molecular mechanism has not been fully elucidated. Existing studies have shown that tumor stemness and epithelial–mesenchymal transition (EMT) play key roles in radiation resistance [25,26,27]. This study focused on the function of the transcription factor TBX18 and proposed that it can form a positive feedback loop by activating SIX1, thereby maintaining the tumor stemness and EMT states and synergistically promoting radiation tolerance.

As a member of the T-box family, TBX18 plays an important role in developmental biology and has been proven to have the functions of promoting proliferation and migration in various tumors in recent years [10,28,29]. However, previous studies mainly focused on the involvement of TBX18 in tumor growth and metastatic behaviors, whereas its role in regulating therapeutic resistance, particularly radiotherapy resistance, remains largely unexplored. In addition, our research team preliminarily explored the role of TBX18 in the radiotherapy of esophageal cancer in previous studies. This study found that TBX18 was significantly positively correlated with stemness-related genes (such as SOX2 and CD44), suggesting that it may act as a regulator of stemness maintenance. Related studies have shown that tumor stem cells have unique advantages in DNA damage repair [30], adaptation to the hypoxic microenvironment [31], and are often naturally resistant to radiotherapy [32]. This study further verified the functional connection between TBX18 and radiation tolerance by combining with stemness pathway inhibitors, filling the gap in the research of this direction. On the other hand, EMT is a key process for various solid tumors to acquire invasiveness and drug resistance. Our study found that TBX18 can also positively regulate EMT markers, and its functional dependence was further confirmed by EMT activator reversal experiments.

It is worth noting that, at the mechanistic level, we found that TBX18 can transcriptionally activate the expression of SIX1 and form a positive feedback regulatory loop with it. SIX1 is a development-related transcription factor and has been widely considered as an important driver of tumor stemness and EMT in recent years. Guo et al. reported that the upregulation of SIX1 in breast cancer can activate enhancing stemness [12]. Chen et al. also reported the regulatory relationship between SIX1 and E-cadherin in colorectal cancer [14]. Our findings therefore identify TBX18 as a novel upstream transcriptional regulator of SIX1 in ESCC. More importantly, we further found that SIX1 stabilizes TBX18 protein expression by suppressing ubiquitin–proteasome-mediated degradation, thereby forming a positive feedback loop characterized by “transcriptional activation-protein stabilization” dual regulation. Such self-reinforcing regulatory circuits are increasingly recognized as critical mechanisms for maintaining stable malignant cellular states and persistent therapy-resistant phenotypes. Our findings extend the current understanding of TBX18 function by demonstrating that TBX18 is not only a downstream effector of malignant progression but also an upstream regulator that establishes a stable transcriptional network through SIX1 activation.

Importantly, analysis of clinical ESCC specimens further supported the biological relevance of this regulatory circuit. Immunohistochemical staining demonstrated that both TBX18 and SIX1 were significantly upregulated in ESCC tissues compared with adjacent normal tissues, and their expression levels showed a significant positive correlation. These findings were highly consistent with our in vitro and in vivo observations and further strengthened the clinical significance of the TBX18/SIX1 axis in ESCC progression and radioresistance. Consistent with previous studies in squamous malignancies, immunohistochemical evaluation of tissue biomarkers may provide valuable information for tumor characterization and clinical stratification. For example, biomarker expression patterns detected by immunohistochemistry have been reported to be associated with prognosis in laryngeal squamous cell carcinoma, highlighting the translational potential of tissue-based biomarker assessment [33]. Therefore, TBX18 and SIX1 may represent potential candidate biomarkers for ESCC, although further validation in larger independent cohorts is required. Moreover, considering the limited effectiveness of current strategies for overcoming radioresistance in ESCC, targeting the TBX18/SIX1 regulatory axis may provide a potential approach to enhance radiotherapy efficacy.

This study has several strengths. First, we combined molecular, cellular, and in vivo approaches to systematically investigate the role of TBX18 in ESCC radioresistance. Second, the reciprocal regulation between TBX18 and SIX1 was demonstrated through promoter analysis, protein stability assays, rescue experiments, and xenograft validation, providing mechanistic evidence for the establishment of this positive feedback loop. Nevertheless, several limitations should be acknowledged. First, although our immunohistochemical analysis demonstrated the clinical relevance of the TBX18/SIX1 axis in ESCC, the number of clinical specimens included in this study was relatively limited. Therefore, the clinical significance and prognostic value of the TBX18/SIX1 axis require further validation in larger independent and multicenter ESCC patient cohorts. Second, although we demonstrated that SIX1 stabilizes TBX18 protein expression, the precise molecular intermediates involved in this ubiquitin–proteasome regulatory process remain unclear and warrant further investigation. In addition, the potential upstream regulators of TBX18, including epigenetic modifications and non-coding RNAs, were not explored in the present study.

## 5. Conclusions

In conclusion, this study demonstrates that TBX18 directly transcriptionally activates SIX1, while SIX1 reciprocally stabilizes TBX18 protein expression, thereby forming a self-reinforcing positive feedback circuit that promotes stemness, EMT, and radioresistance in ESCC. These findings not only expand the current understanding of ESCC radioresistance mechanisms but also suggest that targeting the TBX18/SIX1 signaling axis may represent a promising therapeutic strategy for radiosensitization in ESCC. Future studies should further investigate the upstream regulatory mechanisms controlling TBX18 expression and determine whether pharmacological targeting of the TBX18/SIX1 axis can improve the therapeutic response to radiotherapy. In addition, validation in larger multicenter ESCC cohorts will be necessary to determine its prognostic and predictive value.

## Data Availability

All data which support the findings of the study are within the manuscript or in Supplemental Materials.

## Supplementary Materials

The following supporting information can be downloaded at: https://www.mdpi.com/article/10.3390/cancers18162700/s1, Figure S1. Correlation analysis of TBX18 expression with stemness markers. (A–E) Scatter plots showing the correlation between TBX18 expression and stemness-associated genes (CD44, CD271, SOX2, CD133, and OCT4) in TCGA ESCC samples generated using the GEPIA2 web server. Pearson correlation analysis was performed, and the correlation coefficient (R) and *p* value generated by GEPIA2 are indicated in each plot. TBX18 was positively correlated with CD44, CD271, and SOX2. (F) Western blot validation of TBX18 knockdown (sh-TBX18#1/2/3) and overexpression efficiency in ESCC cells. Relative TBX18 protein levels were quantified by densitometric analysis and normalized to GAPDH. Data are presented as mean ± SD from three independent experiments. ** *p* < 0.01. Figure S2. TBX18 promotes ESCC migration. (A) Transwell migration assays showing reduced migration upon TBX18 knockdown and enhanced migration with TBX18 overexpression. Representative images (left) and quantification (right) are shown. (B) Wound-healing assays at 0 h and 24 h. TBX18 knockdown reduced wound closure, while TBX18 overexpression promoted it. Quantification (right). Data are mean ± SD from three independent experiments. * *p* < 0.05; ** *p* < 0.01. Figure S3. TBX18 cooperates with TGF-β to regulate EMT. (A) Western blot analysis of EMT markers under TBX18 knockdown/overexpression with or without TGF-β treatment. TBX18 synergized with TGF-β to promote EMT. (B) Immunofluorescence staining of TBX18 (red), E-cadherin (green), N-cadherin (yellow), and Vimentin (purple) in ESCC cells with TBX18 manipulation and TGF-β treatment. DAPI (blue) stained nuclei. Scale bar, 20 μm. Figure S4. TBX18 cooperates with TGF-β to regulate irradiation-induced apoptosis. (A) Flow cytometric analysis of apoptosis after TBX18 knockdown, with or without TGF-β, under 0 Gy and 6 Gy. (B) Flow cytometric analysis of apoptosis after TBX18 overexpression, with or without TGF-β, under 0 Gy and 6 Gy. TBX18 overexpression reduced apoptosis, while TGF-β treatment attenuated this protective effect. Data are mean ± SD from three independent experiments. * *p* < 0.05; ** *p* < 0.01. Table S1. Information of the SIX1 promoter fragment used for transcriptional regulation analysis.
