# Supplementary material for: The TBX18/SIX1 Transcriptional Circuit Maintains Stemness and EMT States to Promote Radioresistance in ESCC
Source: Cancers (Basel). 2026 Aug 20;18(16):2700. doi: 10.3390/cancers18162700 (PMC13510439; doi:10.3390/cancers18162700)
Supplement: Supplementary file 1 [file cancers-18-02700-s001.zip › cancers-4427040-supplementary.pdf]

Supplementary Materials

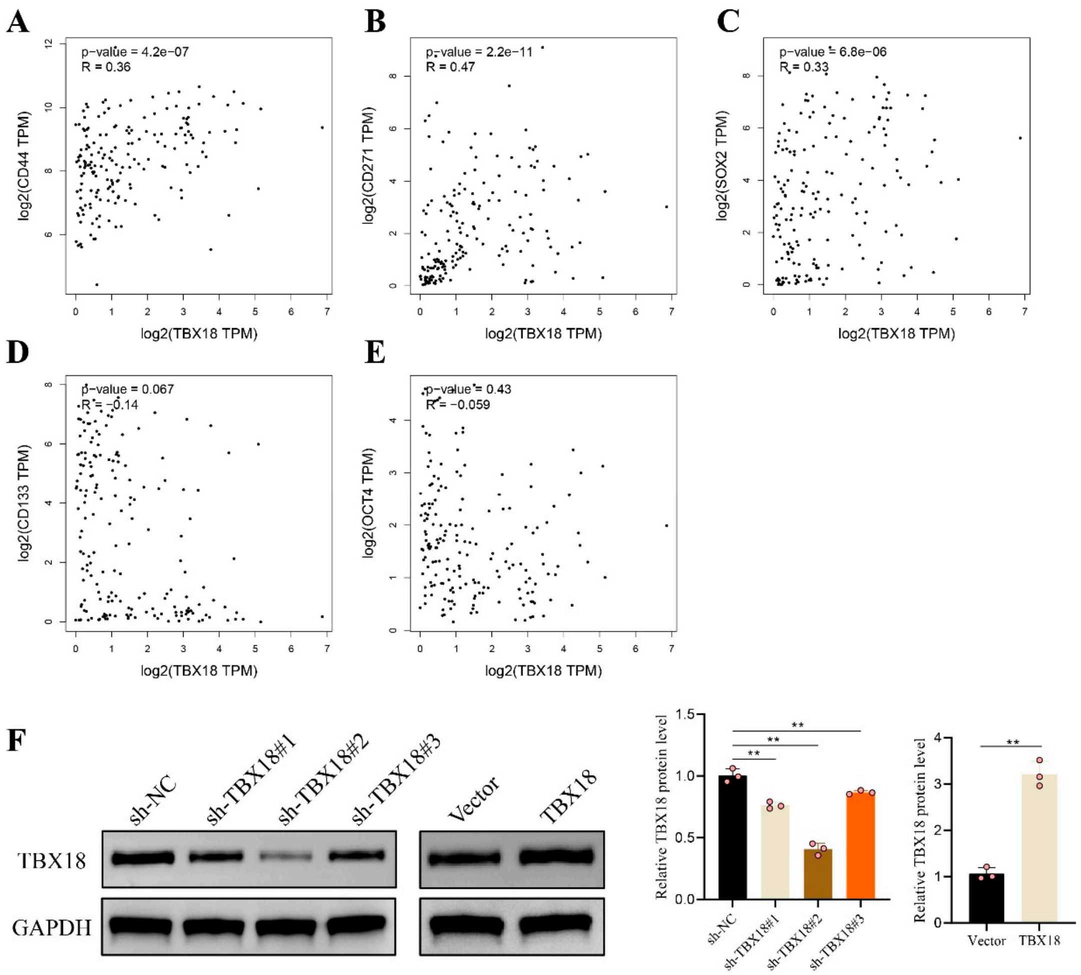

Figure S1. Correlation analysis of TBX18 expression with stemness markers. (**A–E**) Scatter plots showing the correlation between TBX18 expression and stemness-associated genes (CD44, CD271, SOX2, CD133, and OCT4) in TCGA ESCC samples generated using the GEPIA2 web server. Pearson correlation analysis was performed, and the correlation coefficient (R) and p value generated by GEPIA2 are indicated in each plot. TBX18 was positively correlated with CD44, CD271, and SOX2. (**F**) Western blot validation of TBX18 knockdown (sh-TBX18#1/2/3) and overexpression efficiency in ESCC cells. Relative TBX18 protein levels were quantified by densitometric analysis and normalized to GAPDH. Data are presented as mean ± SD from three independent experiments. ** $p < 0.01$.

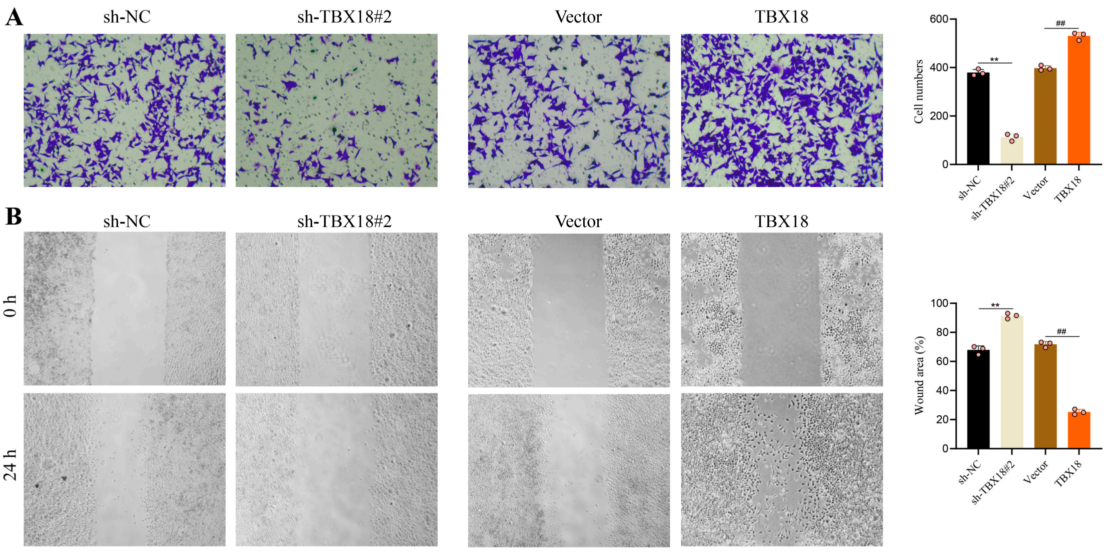

Figure S2. TBX18 promotes ESCC migration. (**A**) Transwell migration assays showing reduced migration upon TBX18 knockdown and enhanced migration with TBX18 overexpression. Representative images (left) and quantification (right) are shown. (**B**) Wound-healing assays at 0 h and 24 h. TBX18 knockdown reduced wound closure, while TBX18 overexpression promoted it. Quantification (right). Data are mean ± SD from three independent experiments. * $p < 0.05$; ** $p < 0.01$.

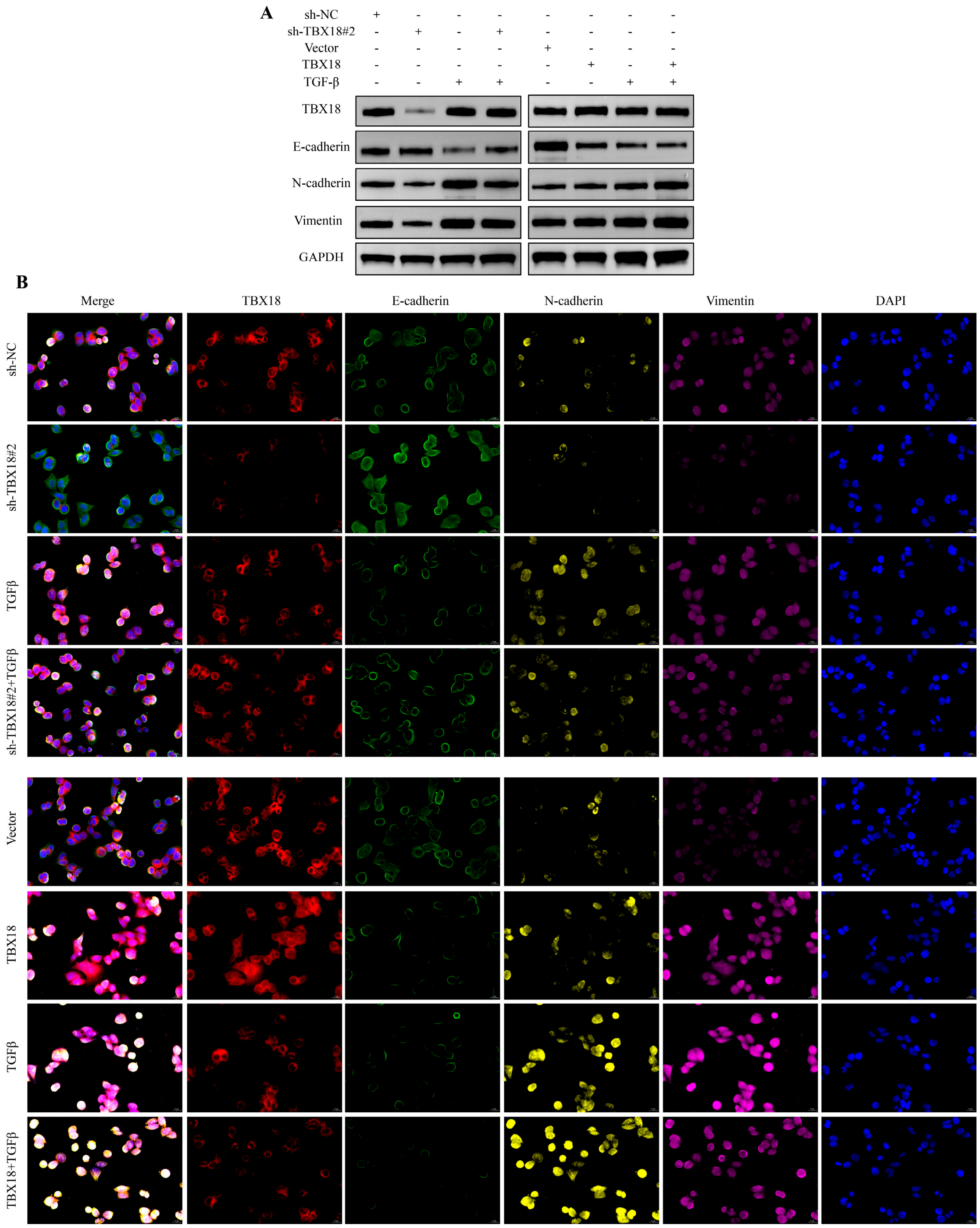

Figure S3. TBX18 cooperates with TGF-β to regulate EMT. (**A**) Western blot analysis of EMT markers under TBX18 knockdown/overexpression with or without TGF-β treatment. TBX18 synergized with TGF-β to promote EMT. (**B**) Immunofluorescence staining of TBX18 (red), E-cadherin (green), N-cadherin (yellow), and Vimentin (purple) in ESCC cells with TBX18 manipulation and TGF-β treatment. DAPI (blue) stained nuclei. Scale bar, 20 μm.

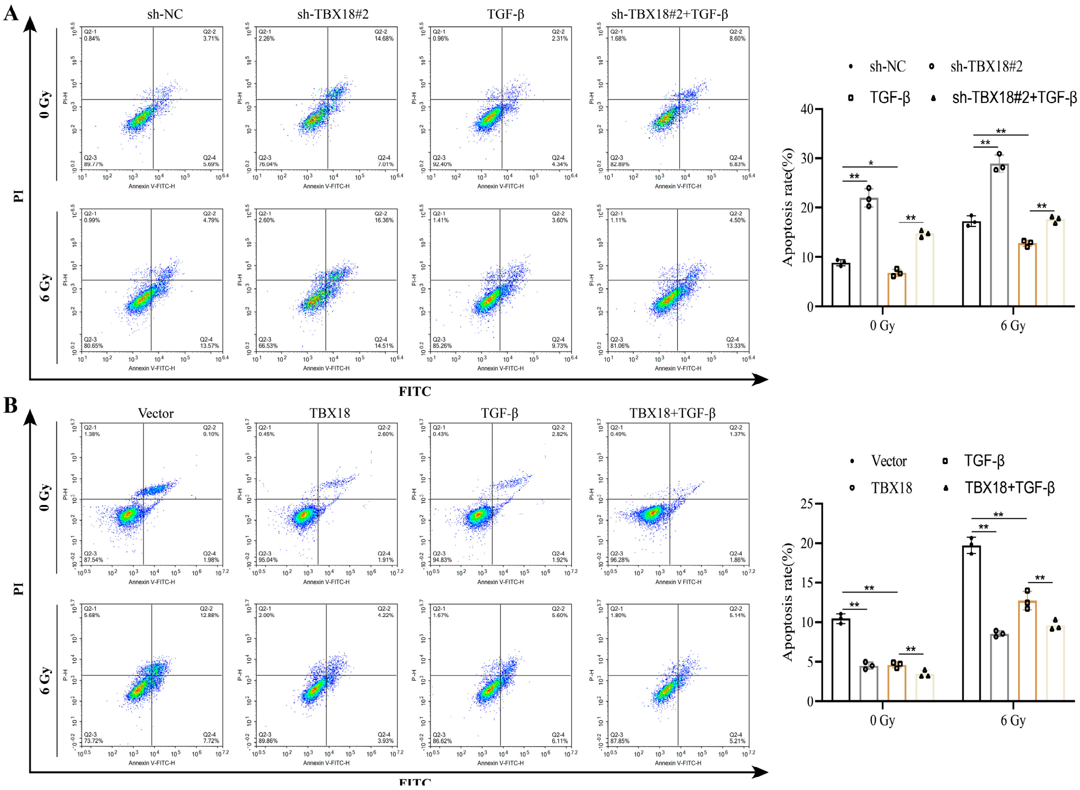

Figure S4. TBX18 cooperates with TGF-β to regulate irradiation-induced apoptosis. (**A**) Flow cytometric analysis of apoptosis after TBX18 knockdown, with or without TGF-β, under 0 Gy and 6 Gy. (**B**) Flow cytometric analysis of apoptosis after TBX18 overexpression, with or without TGF-β, under 0 Gy and 6 Gy. TBX18 overexpression reduced apoptosis, while TGF-β treatment attenuated this protective effect. Data are mean ± SD from three independent experiments. * $p < 0.05$; ** $p < 0.01$.

Table S1. Information of the SIX1 promoter fragment used for transcriptional regulation analysis.

Gene	Genome assembly	Region	Length
SIX1	GRCh38.p14	NC_000014.9:c60651477-60649477	2001 bp

Promoter sequence (5′→3′):

>NC_000014.9:c60651477-60649477 Homo sapiens chromosome 14, GRCh38.p14 Primary Assembly

TTTAGAAAGTCATTGGGCATACTATATGGCAGAGGCTCTTTTGAAAGCATGCACACCTA
TTAAATGCATTTTTTTAAAAAAAAAGCCTAAGGTAATGAAATATCAGCATCTGCTTCCA
AGATCTAATTGCATGGAATAAGCAAGAGTTTCAAGGATGCAGAAAACATTATGTTCAGC
AGAGAAGCAGCTTCCTGTCTACTTGGAATCTCCCATGTGGCAGCCCTGAGGTGGGAAC
CCTTTATGAGTTTAGAATGTATGTTGCAGGGTGCACTGTACTGGGTGCATCATTTTAAAA
CCATATTAATAATAAAAGAGGAAACCAAGACATGCTTCTCCTTCTCCCACTTGCATCTC
AATATGCAAAAGGTTCTGTAGTGCTGGGCTCTTTGGCTAAAGGAATCAAAACATATTTG
AAGAAGACAAGCCAGTTTAAGGAGCCTACAGTTTGTTCTTCACCACTCACTAAGAGGG
CACATTGAAATCAAAAGCATTTTTGAAGTGTACTTAGAATTCTGTTTTGAACTTATCTAA
TCAGAGATAAGTATAGTCATAAAGTCACTCTCCTTCCACCATTTGGCTTCCCTTATCTCC
ACGCATTGTTCTTATCTCCTGCTAGTCTCAGCTACTGACAATAGATTATCTTTGTTTAGA
GAGGCTAATTGTTTTCAAAATCCTTTTAATTATTGCTTTGGAACTAGGTGTGCAAATGAG
GCTTAAGATAATTACAACTTCACTTGTTTGTGCAGTTTTACTAAGCATTCTCCACACAT
AAACAAAACAGCAGATGATGGGACTTAAATTGCAGTGCATCTCTCATTCCCTGGCAGC
AAAATAGGCCAGCATTGCAACACACACACACACACACATACACACACACACACAC
ACACACACCTCTTTATTTAATCTTCTTGGAGCCGCTGTCGTTCTCGCTTTGAGGGCAGT
GTCCCTCTCAGAGCAGGGTAAGGATTATATTACAACCGGACGGTTATTGGAGCCGGGC
ACAGTTTTATTTAATTTACACCACATCCAGCGCCTTTCGTACGAGGTTCCCAAAGCATTT
TTGCTAATAGCAAGTAAAGCCTCTCGGCATTCCTCAGTAGCAGAGGTGGCACTGCAAA
ACACAACCGCACAGATGGGGAAATTGAGTCCAGAAGCTTCAGGAACCTGCTGGTTTC
AATCGCAGAACATTAAGATACCAAATCACAAATGAACGCTCGGAGTCTCTAGGTGCTC
AGCAAGTATGCTCTAAACAGCTCTCTGCCTGGTCTCAAATCATGGAGGTCTTTTGTGAA
CGAGGGAAACGCTGTCCGACTGGGAGTACTTGTGCCCTTTGGAGGGTGGTGTGCAGC
AGTGGGGGGAGGACAGTGGTAGTTGGTGGGGGGAGGGAGGAGGAAATAGGGTTGGA
CTCCTCGAGCTGAAGCCAACCCGAAGCCACTCTTCCAGAGGCTGCATGCGCGGACCCT
GGTCTGCTCTTGAATTATTTATCGCGTTCCATAAGCTATTCATCAATCCATTATTTATTCAT
TCCAACATTAAAGTAATTAAATATGAGCTCACCGGGAGAGGGCAAGTCTGCAAGGGAT
GAGGACAAGAGTACGGAGGGAGAGGGGAGGAATTAAGGTTTGGAGTCATTGATTTGT
GCGGAGTTGTGCGCGACTCCCCTTAGGTGATACTGGCGACGGGTCCCGCGTTGCAGGG
TCCTGACGCGCTCACCCACTCCCTACCTCCCCACACCTCCGCAGCGCAGCTCCCTCAG
ACCCCAACTGGCTTCCCACCTTTCCTCTTCCTTCTCCTCCCACCACTCCCTACCCGCGC
AGCCCCGGCAACACCCCCAACCCCCAGCTCCGCAGCCTCGCCCTCCCCGCTCTGGGCG
GTGCGGCCTCTCTGCCCAGCCGGGCGGCGCGCTGGCTGGCCAGCCCCGGATAGGCGC
CGCCGGCAGCCAATCAGCGTGCCGGGGACGCTGCGCGCTTTAAGGCCGCTGCGGGGA
GAACAGAAACCA
